# A New Strategy in Boosting Information Spread

**DOI:** 10.3390/e24040502

**Published:** 2022-04-02

**Authors:** Xiaorong Zhang, Sanyang Liu, Yudong Gong

**Affiliations:** 1 School of Mathematics and Statistics, Shaanxi Xueqian Normal University, Xi’an 710061, China; 2 School of Mathematics and Statistics, Xidian University, Xi’an 710071, China; liusanyang@126.com (S.L.); gongyd.up@foxmail.com (Y.G.)

**Keywords:** realistic propagation model, boosting information spread, B-degree discount algorithm

## Abstract

Finding a seed set to propagate more information within a specific budget is defined as the influence maximization (IM) problem. The traditional IM model contains two cardinal aspects: (i) the influence propagation model and (ii) effective/efficient seed-seeking algorithms. However, most of models only consider one kind of node (i.e., influential nodes), ignoring the role of other nodes (e.g., boosting nodes) in the spreading process, which are irrational. Specifically, in the real-world propagation scenario, the boosting nodes always improve the spread of influence from the initial activated seeds, which is an efficient and cost-economic measure. In this paper, we consider the realistic budgeted influence maximization (RBIM) problem, which contains two kind of nodes to improve the diffusion of influence. Facing the newly modified objective function, we propose a novel B-degree discount algorithm to solve it. The novel B-degree discount algorithm which adopts the cost-economic boosting nodes to retweet the influence from the predecessor nodes can greatly reduce the cost, and performs better than other state-of-the-art algorithms in both effect and efficiency on RBIM problem solving.

## 1. Introduction

Social network is the structured representation of authentic network relations, which has attracted the widespread attention of researchers around the world [1,2,3]. The IM problem is a key problem in social networks, and its aim is to find a influential nodes set whose influence spreading is maximized [4]. It is widely used in collaborative filtering, political analysis, link prediction, web search and recommendation systems [5,6,7,8,9].

In order to solve the IM problem, Domingos et al. summarized it as an optimization model firstly [10]. Then IM was generalized as a mathematical problem by Kempe et al., and two propagation models were proposed [11]: independent cascade (IC) model [12,13,14] and linear threshold (LT) model [15,16]. They proved that find the optimal solution of such a problem is NP-hard, and two approximate methods (one greedy and one heuristic) were proposed to solve it. Generally, the IM problem mainly contains two core parts: diffusion models and the selection method of the initial node set. Except the IC and LT models, Ganesh et al. proposed the epidemic model [17,18], which uses the graph’s topological properties to simulate the persistence of epidemics. Tzoumas et al. proposed a game-theoretic model [19,20] which using the known linear threshold model to simulate the diffusion of 2-player games. Meanwhile, some greedy algorithms [21,22,23], heuristic algorithms [24,25] and their extensions [26,27] have been presented to find the most influential seed sets. Leskovec et al. [28] proposed cost-effective lazy forward selection (CELF), which, according to the sub-modularity of the influence maximization objective, achieves near-optimal placements. Chen et al. proposed the NewGreedyIC algorithm, which can decrease the time costs and optimize the diffusion of influence [23]. Gong et al. [27] proposed the memetic algorithm, and designed population initialization and local search to improve the algorithm efficiency.

To simulate more realistic propagation scenarios, Lin et al. proposed boosting the influence model firstly, which selected the boosting nodes set to increase the influence spread of the initial seed nodes [29]. Shi et al. further proposed a new framework which gave the cost of seed/boosting nodes and considered the optimal nodes set in a social network with a constrained budget [30]. However, these works did not clarify the different influence between seed nodes and boosting nodes.

In this paper, a more flexible budget model is proposed to improve the shortcoming of propagation models, and a new nodes selection strategy is proposed to improve the propagation efficiency of the new model. This paper’s contributions are summarized as follows:

(1) We propose a new framework for influence maximization (RBIM) for specific scenarios to distinguish the different influence between seed nodes and boosting nodes.

(2) We propose a new strategy which first looks for candidate boosting nodes and then reverse finds seed nodes that have less influence.

(3) We introduce a new strategy based on the degree discount algorithm, so that the degree discount algorithm can iteratively select seed nodes and boosting nodes.

## 2. Related Work

Given a graph G(V,E), where $V=(V_{1},V_{2},\ldots ,V_{n})$ is the set of all nodes, *E* is the set of all edges. The IM problem’s aim is to find most influential nodes set within the budget *k* [31]. We can generalize it as a constrained optimization problem:

(1) $$S*=\underset{S\subseteq V}{argmax}\sigma (S),s.t.C\left(S\right)=k,$$

where *S* is the selected set of seed nodes, σ(S) is the expected final influence of the nodes in *S*, and C(S) is the expected cost of *S*.

**Figure 1:**
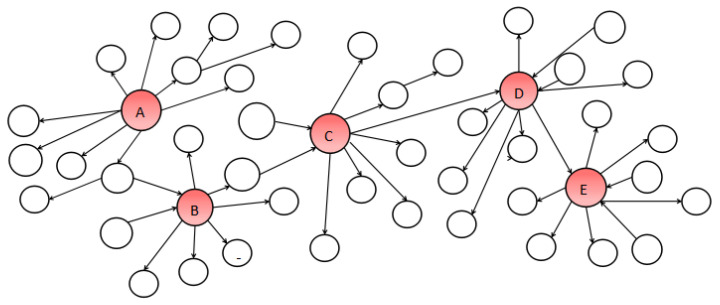
Example of IM problem.

Figure 1 is a simplified network diagram of the IM problem, each node’s cost is 1. The IM problem’s aim is to find the optimal set *S*. We choose the most influential node as the seed node. When the budget *k* is 1, we choose *A* as the seed node. When *k* is 2, the seed nodes set can be (A,C), (A,B) or (A,D).

Equation (1) is mainly made up of two parts: propagation models and selection method of initial nodes. In the development of social networks, numerous models have been proposed to simulate information diffusion process. Kempe et al. [11] proposed two classical diffusion models firstly: (i) The independent cascade (IC) model [32] supposes that a user *v* can be activated by its predecessor *u* with probability $p_{\left(u,v\right)}$ through edge $e_{\left(u,v\right)}$. (ii) The linear threshold (LT) model’s basic idea is that a user *v* can be activated when it has a sufficient number of predecessor nodes in the actively status. Besides the IC and LT models, the epidemic model [17,18] and game-theoretic model [19,33] have also been devised to simulate the process of information diffusion. In order to find influential nodes, some greedy algorithms [21,22,23], heuristic algorithms [24,25,34], and their extensions [26,27] have been proposed. Leskovec and Krause [11] proposed the CELF algorithm, which utilizes the sub-modularity of the model to find the near-optimal solution in a sparse large network. Furthermore, Goyal [22] proposed a highly optimized approach based on the CELF algorithm, which uses the property of sub-modularity. Besides the greedy algorithm, a degree discount heuristic algorithm was proposed [23], which uses the degree to measure the influence of nodes. Roaa et al. proposed a new degree discount heuristics that improves the influence spread [35]. Kitsak et al. [36] proposed the coreness/location as an important index to determine the node spreading, which is named the k-shell algorithm. He et al. [37] proposed the two-stage iterative framework, which uses the iterative framework to select the candidate nodes set, and removed the apical dominance to select the final seed nodes.

With the popularity of internet propagation scenarios, the boosting influence spread model began to attract scholars’ attention. Lin et al. first proposed the *k*-boosting problem, which selects appropriate boosting nodes to increase the influence diffusion of initial seed nodes [29]. Then, Shi et al. [30] further proposed holistic budgeted influence maximization, which uses a new framework based on the boosting influence spread model. In this article, the author maximized the influence spread by overall planning the cost of seed nodes and boosting nodes in a constrained budget. However, these works do not specifically divide the influence of the seed node and boosting node. Actually, we can comprehensively consider the influence and cost between seed and boosting nodes, which is more practical in a social network.

## 3. Proposed Methodology

**Figure 2:**
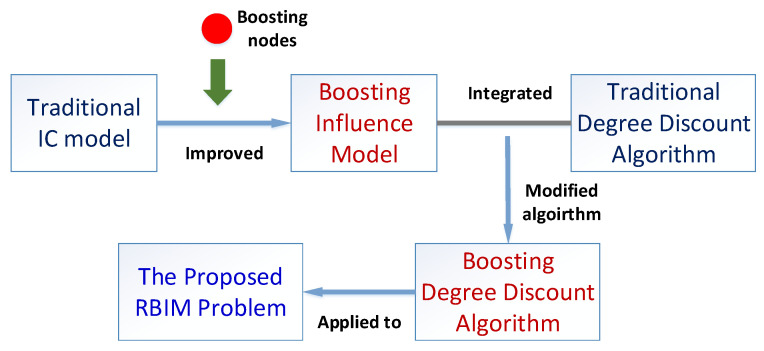
The diagram of the proposed methodology.

In this paper, instead of studying the traditional influence maximization (IM) problem, we consider a novel realistic budgeted influence maximization (RBIM) problem which aims to find both seed nodes and boosting nodes. Figure 2 illustrates the main framework of the proposed methodology. Firstly, the boosting nodes are introduced into the IC model, and the influence diffusion process is improved for a more realistic scenario—the boosting influence model. Then, the traditional degree discount algorithm is adopted, and a modified boosting-degree discount method is proposed, which can achieve efficient and effective results on RBIM problem solving.

**Definition** **1** (Independent Cascade Model [29]). *In a graph G=(V,E), there is an edge $e_{\left(u,v\right)}\in E$ between two nodes u and v. The newly activated node u can activate node v with probability $p_{\left(u,v\right)}$. The aim of the IM problem is to find the most influential seed nodes set with a constrained budget k.*

### 3.1. Boosting Influence Model

**Definition** **2** (Boosting Influence Model [30]). *In a graph G=(V,E), there is an edge $e_{\left(u,v\right)}\in E$ between two nodes u and v. The newly activated node u can activate node v with probability $p_{\left(u,v\right)}$, and can boost node v with probability $p_{\left(u,v\right)}^{{}^{\prime }}$$\left(p_{\left(u,v\right)}^{{}^{\prime }}>p_{\left(u,v\right)}\right)$. The aim of the boosting influence model is to find the most influential seed nodes and boosting nodes set with a constrained budget k.*

Definition 2 proposed the boosting influence model. A group of nodes is defined as boosting nodes. These boosting nodes would receive and propagate information from the predecessor nodes with a higher probability. For example, people are more willing to forward a tweet that was published by their friends. We can choose users who have less influence and use a lower cost (such as trial and discount) to persuade them to publish a given article. The boosting nodes we selected are more easily affected by their friends with a specific probability.

### 3.2. Realistic Budgeted Influence Maximization Problem (RBIM)

**Definition** **3.** RBIM problem: given a graph G=(V,E), the aim of the RBIM problem is to find a set (S,B)* which can achieve the maximize influence with a constrained budget k:

(2) $$\left(S,B\right)*=argmax_{\left(S,B\right)}\sigma \left(S,B\right),s.t.C_{s}\left(S\right)+C_{b}\left(B\right)\leq k,$$

where *S* denotes the initial seed set, and *B* denotes the initial boosting node set, σ(S,B) represents the expected influence of the binary (S,B), $C_{s}\left(S\right)=\sum_{u\in S}$$c_{s}\left(u\right)$ and $C_{b}\left(B\right)=\sum_{u\in B}c_{b}\left(u\right)$$c_{s}\left(u\right)$ and $c_{b}\left(u\right)$ represent the total costs of nodes in *S* and *B*, respectively. In addition, it is noted that the cost of activating a node as a seed or a boosting node is different, and generally, an individual prefers to transmit the message than propose firstly, so we set costs of both to satisfy $c_{s}\left(\cdot \right)\gg c_{b}\left(\cdot \right)$ for each node. Meanwhile, we give the cost for the seed node, the propagation probability and boost probability between each of the two nodes. It is reasonable to assume that if a node is selected as a boosting node, its influence propagation probability is lower than when selecting it as a seed node (for example, v∈V, $\sigma_{B(v)}=0.8\times \sigma_{S(v)}$).

**Figure 3:**
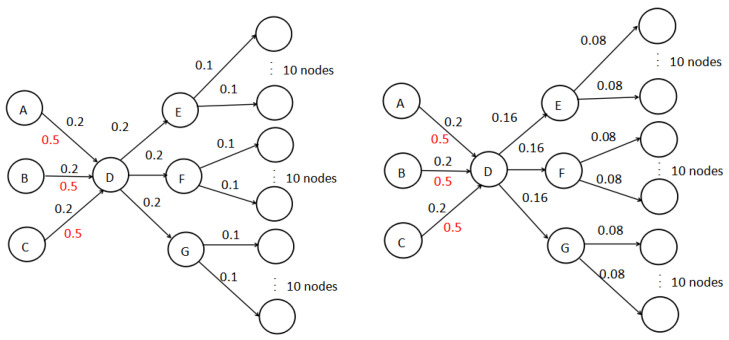
The diagram of RBIM.

We drew a diagram to illustrate RBIM problem in Figure 3. In the propagation diagram, the black value represents the propagation probability and the red value represents the boosts probability. Table 1 lists the cost of each node in Figure 3. The diagram on the left is the propagation influence diagram with node *D* as the seed node, while the diagram on the right is the propagation influence diagram with node *D* as the boosting node. The solution of the IM problem is to select *A* and *B* as seed nodes with budget (k=2) and its’ expected influence spread is 0.88 (the seed nodes is not calculated in the expected influence spread). However, the RBIM problem has a better solution with the same budget. We can select *C* as the seed node and *D* as the boosting node; the influence spread is 1.1.

### 3.3. The New Strategy

**Figure 4:**
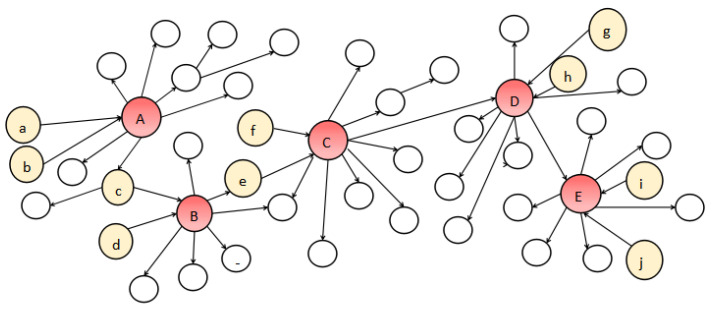
The example of new strategy.

We compare the traditional strategy with the new strategy in Table 2. Figure 4 is a simplified diagram of a small data network. In a real social network, nodes with different influence usually have a different cost. In this example, nodes with more than five successors are considered high influence nodes and others are considered low influence nodes. The cost of the high influence node as the seed node is 1, and the cost of the boosting node is 0.5. The cost of the low influence node is 0.1. We choose the node with the largest number of successors and judge whether it is the seed node or the boosting node according to probability. In the above schematic diagram, we first select node *C* as the candidate node according to the number of successors, and select seed node *f* from the predecessors of node *C* to boost node *C* with a probability of 0.6. If *C* is successfully boosted, S=(f),B=(C), $C_{s}\left(S\right)=0.1,C_{b}\left(B\right)=0.5$. If it is not boosted successfully, we continue to select node *e* to boost node *C*. If node *C* is boosted successfully, $S=\left(f,e\right),B=\left(C\right),C_{s}\left(S\right)=0.2,C_{b}\left(B\right)=0.5$; otherwise $S=\left(f,e,C\right),C_{s}\left(S\right)=1.2$. Under this strategy, we find that the expected cost of the boosting node *C* is E(cost(c))=0.6×0.6+0.24×0.7+0.16×1.2=0.72. We can see that the expected cost is lower than the cost of directly selecting seed nodes without using this strategy. However, in real-life scenarios, the cost of high influence nodes is much higher than that of low influence nodes. Using this strategy can save expenses effectively (obtain greater influence within the same expenses).

### 3.4. Improved Degree Discount Algorithm Introduction

In this subsection, we improve the degree discount algorithm. The primary idea of the degree discount is that when *v* is activated, then the degree of all its neighbors should not count their edges linked to node *v* (i.e., $d_{u}^{{}^{\prime }}\to d_{u}-1$, u∈N(v)(N(v) express the neighbors set of node *v*).

The cost of selecting a node as a boosting node is much lower than that of activating it as a seed node in realistic scenarios. In today’s internet media era, the influence of media’s original works is often greater than the works obtained from the third-party platform in the fixed communication network. For example, the cost of employing a hub agent to forward an advertisement message is relatively lower than asking him to publish the original message. We can choose influential person’s friends to boost an influential person with a lower cost.

**Figure 5:**
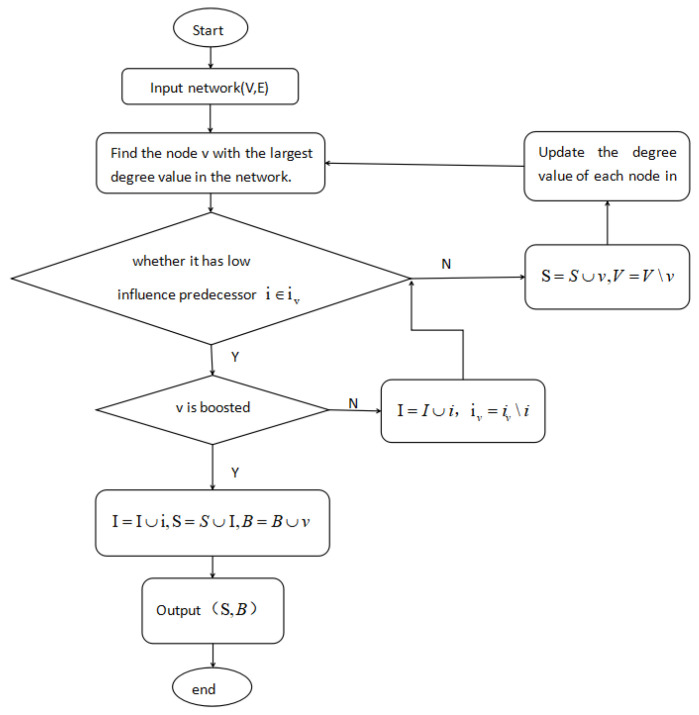
Algorithm flow chart.

Figure 5 shows the flow chart of the proposed boosting degree discount algorithm. The main iteration process is as follows: firstly, select the node *v* with the greatest influence within the budget as the candidate node and judge whether *v* can be effectively boosted by the predecessor node with low influence; then, update seed nodes set *S* and boosting nodes set *B* according to the boosted state of *v* within the budget.

In this section, we propose the RBIM problem based on the real network influence propagation model. In order to further solve the RBIM problem, we propose a node configuration strategy which uses low-cost nodes to boost highly influential nodes. The strategy is integrated into the degree discount algorithm. The detailed process of boosting the degree discount algorithm is described in Algorithm 1: firstly choosing a candidate boosting node according to the degree of nodes; secondly, reverse find the seed nodes through boosting node which is the candidate boosting nodes’ predecessors; lastly, calculate the cost of the boosting node and its seed nodes to decide whether to leave it in boosting nodes set *B*. In the specific setting of the algorithm, it is reasonable to assume that the cost of seed node *v* is closely related to the degree $d_{v}\left(c_{s}\left(v\right)=\phi \left(d_{v}\right)\right)$.
**Algorithm 1** *B*(Boosting)-degree discount algorithm (G,k). initialize S=ϕ,B=ϕ (*S* represents the seed nodes set, and *B* represents the boosting nodes set) **for** each node *v* **do**    calculate the degree $d_{v}$    $c_{S}\left(v\right)=\phi \left(d_{v}\right)$    $c_{B}\left(v\right)=0.5\times c_{S}\left(v\right)$    compute its input degree $i_{v}$ **end for** **for** i in (1:*k*) **do**    select $u=argmax_{v}\left(d_{v}|v\in V\backslash S\cup B\right)$    $S_{u}=Ø$    **for** *i* in $i_{u}$ and $d_{i}<0.1\times d_{u}$ **do**      $q_{u}=q_{u}\times \left(1-p_{iu}\right)$      $c_{B}\left(u\right)=c_{S}\left(i\right)+c_{B}\left(u\right)$      $S_{u}=S_{u}\cup i$      **if** $q_{u}<0.05$ **then**         **if** $c_{B}\left(u\right)<c_{S}\left(u\right)$ **then**           $S=\left\{S⋃S_{u}\right\},B=\left\{B⋃u\right\}$,         **else**           S={S⋃u}           $c_{B}\left(u\right)=0.5\times c_{S}\left(u\right)$           break         **end if**      **else**         continue      **end if**    **end for** **end for** **if**
*u* in *S* **then**    **for** each neighbor v∈V\S of *u* **do**      $t_{v}=t_{v}+1$      $dd_{v}=d_{v}-2t_{v}-\left(d_{v}-t_{v}\right)t_{v}p$    **end for** **else**    **for** each input degree *m* of *u* **do**      **for** each neighbor *n* of *m* **do**         **if** n≠u **then**           $t_{n}=t_{n}+1$           $dd_{n}=d_{n}-2t_{n}-\left(d_{n}-t_{n}\right)t_{n}p$         **end if**      **end for**    **end for** **end if**


## 4. Experiments

### 4.1. Data Sets

We test the performance of the new algorithm on synthetic data sets and real-world data sets. Table 3 lists the characteristics of the tested data sets. The three synthetic data sets are processed from three classical data sets, namely, ER-directed graph [38], BA-directed graph [39] and WS-directed graph [40]. Because the new strategy needs to consider the predecessor and successor nodes of candidate nodes, we modify three classical synthetic networks as follows. Firstly, we set the number of nodes n=3000, the probability of the edge generation between two nodes as 0.01, and generate the ER graph. Then, we convert it into a directed network and the edge is deleted randomly with a probability of 0.6. The ER-directed network includes 3000 nodes and 35,690 edges. We generate the BA network by setting the number of nodes n=3000, the number of edges m=10 for each node. Each new node generated in the network needs to establish *m* edges with the existing nodes until all nodes are generated. Then, we convert it into a directed network and the edge is deleted randomly with a probability of 0.6. The BA-directed network includes 3000 nodes and 23,837 edges. We generate the WS network from a circular network containing 3000 nodes and each edge in the network is randomly reconnected with a probability of 0.05. Then, we transform the WS network into the directed network and delete edges randomly with a probability of 0.6. The WS-directed network includes 3000 nodes and 23,993 edges. We adopt three real-world network data sets in the experiments. The detailed characteristics of the three network data sets are as follows. Epinions is a consumer review web site based on mutual trust. Web site members can independently decide whether to trust each other, then build a trust network through a trust relationship. This network consists of 75,819 nodes and 508,836 edges (http://snap.stanford.edu/data/soc-Epinions1.html, (27 September 2021)). Wiki-Vote is the voting data of Wikipedia administrators, which establishes social networks through voting and being voted. The network include 7115 nodes, 103,689 edges (http://snap.stanford.edu/data/wiki-Vote.html, (30 September 2021)). The DBLP site is a reference network of scientific publications, which is constructed by the reference of each publication to another publication. It includes 12,591 nodes and 49,743 edges (https://dblp.uni-trier.de/db (1 October 2021)).

### 4.2. Baseline Algorithms

We compare the new algorithm with five classical baseline algorithms. The basic ideas of these five baseline algorithms are as follows:

Iv-greedy: Iv greedy calculates the influence of each node, repeatedly selects the node with the largest marginal influence [41].

celf: The celf’s core idea is that, with the increase in selected nodes, the marginal influence of each node can never increase. Its iterative process is as follows: Initially, the non seed nodes set are arranged in descending order according to the marginal influence. When a new seed node appears, we recalculate the marginal influence of the top element of the non seed nodes sequence. Generally, the marginal influence of the top node is still largest and arranged at the top, so as to reduce the time complexity [28].

Degree: The core idea of the degree algorithm is to calculate each nodes’ degree and select the seed nodes from the nodes which have a large degree [11].

Single degree discount: The core idea is to discount each neighbor of the newly selected seed.

Degree discount: The degree discount algorithm is based on the degree algorithm to remove the degree value of the degree node that is entered as the seed node and constantly update the degree value of the non seed node [42].

### 4.3. Experiments and Results

We conduct experiments on three synthetic networks and three social network to show the effectiveness of the new algorithm. The code is written in Python, and all experiments are tested on a laptop with Intel (R) core (TM) I5-10300h CPU with 2.5 GHz and 16.0 GB memory under Windows 10 64-bit operating system.

We show the experimental results of six network data sets in the figure below. Each curve shows the change of influence diffusion relative to constraint budget *k*. In the experiment of this paper, we set the budget *k* from 1 to 20, the activation probabilities of these network seed nodes are 0.01, 0.01, 0.001, 0.02, 0.05, and 0.1.

**Figure 6:**
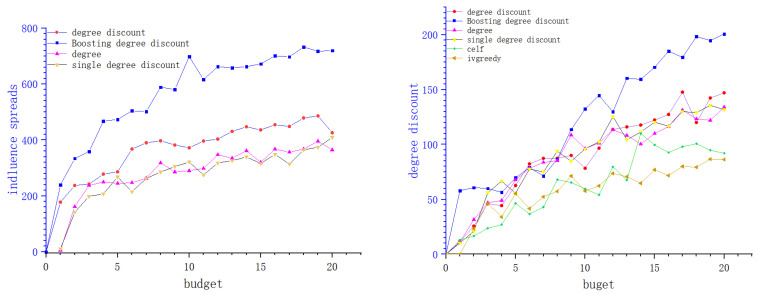
Epinions and Wiki.

**Figure 7:**
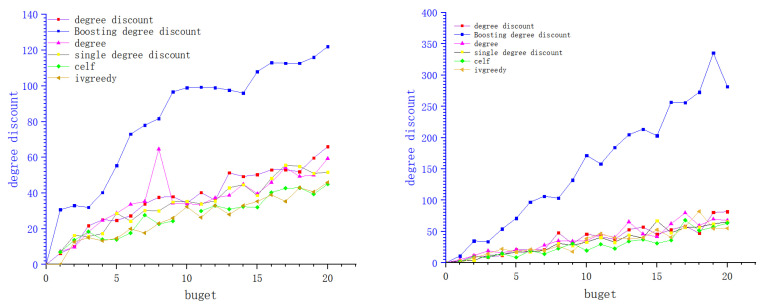
Dblp and ER_to_directed.

**Figure 8:**
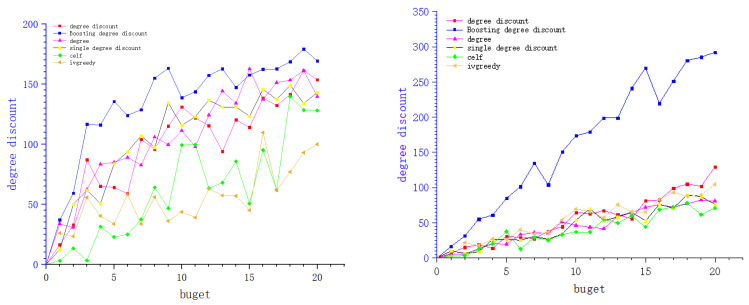
BA_to_directed and WS_to_directed.

In this part of the experiment, we simplify the model and set the cost of the boosting node as 0.5, then the corresponding influence is reduced to 0.8 of the original activation probability, while the cost, when it is regarded as a seed node, is 1. In the boosting degree discount algorithm, we inversely find seed nodes which can activate the boosting node, and the boost probability is 0.5. In the original hypothesis, we set the reverse search seed node as the node with relatively small influence (in the microblog scenario, we find the blogger with a large number of fans as the boosting node, and the blogger with a small number of fans is the seed node according to the crowd concerned by the blogger). We set its cost as 0.05 if the number of successor nodes do not exceed 0.05 of the number of successor of the boosting node. In Figure 6, Figure 7 and Figure 8 of this article, each picture represents a data set. The *X*-axis of the picture represents the given budget, the *Y*-axis represents the influence that can be achieved, and the different curves on each picture represent different algorithms. From the experimental results, it can be seen that under the same budget conditions, the boosting degree discount algorithm can obtain greater influence. In summary, the boosting degree discount algorithm is better than other algorithms in our realistic model.

## 5. Conclusions

This paper proposes a more realistic propagation model, which comprehensively considers the cost and influence of different role nodes. Based on this scenario, we propose a new strategy, which uses low-cost nodes to activate nodes with high influence. We introduce the above strategy into the traditional degree discount algorithm so that the degree discount algorithm can iteratively select the optimal node set for the seed nodes and the boosting nodes under the condition of the lowest average marginal cost. The results of the experiment show that the improved degree discount algorithm has greater influence under the same budget. In future work, we can divide the cost of each node according to its expected influence (or other measurement indicators such as degree) to make it more in line with real-life scenarios. The strategy of finding the boosting node first and finding the seed node in reverse can be combined with other propagation model algorithms to solve the influence propagation problem with a more flexible strategy.

## Figures and Tables

**Table 1:** The cost of nodes.

	*A*	*B*	*C*	*D*
cost (seed)	1	1	1	3
cost (boost)	0.3	0.3	0.3	1

**Table 2:** Comparison of new strategy and traditional strategy.

	New Strategy	Traditional Strategy
role of node	seed node, boosting node	seed node, boosting node
relationship between different role nodes	The seed node is the predecessor of the boosting node	no connection
advantage	The cost is low and the role division of different nodes is obvious	The node iteration process is simple

**Table 3:** Nodes set.

Data Sets	Nodes	Edges	Average Degree
ER_to_directed	3000	35,690	11.85
BA_to_directed	3000	23,837	7.95
WS_to_directed	3000	23,993	8.0
Epinions	75 k	508	6.70
Wiki-Vote	7 k	103 k	14.57
Dblp	12 k	49 k	3.95
